# Activated Notch Causes Deafness by Promoting a Supporting Cell Phenotype in Developing Auditory Hair Cells

**DOI:** 10.1371/journal.pone.0108160

**Published:** 2014-09-29

**Authors:** Grace Savoy-Burke, Felicia A. Gilels, Wei Pan, Diana Pratt, Jianwen Que, Lin Gan, Patricia M. White, Amy E. Kiernan

**Affiliations:** 1 Department of Ophthalmology, University of Rochester Medical Center, Rochester, New York, United States of America; 2 Department of Biomedical Genetics, University of Rochester Medical Center, Rochester, New York, United States of America; 3 Department of Neurobiology and Anatomy, University of Rochester Medical Center, Rochester, New York, United States of America; Instituto de Medicina Molecular, Portugal

## Abstract

**Purpose:**

To determine whether activated Notch can promote a supporting cell fate during sensory cell differentiation in the inner ear.

**Methods:**

An activated form of the Notch1 receptor (NICD) was expressed in early differentiating hair cells using a Gfi1-Cre mouse allele. To determine the effects of activated Notch on developing hair cells, Gfi1-NICD animals and their littermate controls were assessed at 5 weeks for hearing by measuring auditory brainstem responses (ABRs) and distortion product otoacoustic emissions (DPOAEs). The differentiation of NICD-expressing hair cells was assessed at postnatal day (P) 6, 11 and 20, using histological and molecular markers for hair cells, as well as supporting cells/progenitor cells. We also examined whether the effects of Notch were mediated by SOX2, a gene expressed in supporting cells and a likely downstream target of Notch, by crossing an inducible form of SOX2 to the Gfi1-Cre.

**Results:**

Activation of Notch1 in developing auditory hair cells causes profound deafness. The NICD-expressing hair cells switch off a number of hair cell markers and lose their characteristic morphology. Instead, NICD-expressing hair cells adopt a morphology resembling supporting cells and upregulate a number of supporting cell markers. These effects do not appear to be mediated by SOX2, because although expression of SOX2 caused some hearing impairment, the SOX2-expressing hair cells did not downregulate hair cell markers nor exhibit a supporting cell-like phenotype.

**Conclusions:**

Our data show that Notch signaling inhibits hair cell differentiation and promotes a supporting cell-like phenotype, and that these effects are unlikely to be mediated by SOX2.

## Introduction

The vertebrate inner ear is a complex structure that includes a variety of sensory regions that transduce both sound and vestibular information. Each sensory region is composed of two major cell types, the sensory hair cell and associated supporting cells, which arise from a common progenitor [1]. The mosaic arrangement of the sensory cells, in which each hair cell is surrounded by supporting cells, led investigators to suggest that the pattern was produced through the process of lateral inhibition mediated by the Notch signaling pathway [2], [3]. Notch signaling is an evolutionarily conserved pathway in which interactions between the cell-bound ligands (Jagged1–2, and Delta-like1,3–4) and receptors (NOTCH1-4) trigger the release of the activated form of the receptor (the intracellular domain or NICD) to the nucleus where it interacts with the nuclear effector RBPJ (also known as RBPjκ or CSL) and causes changes in transcription (reviewed in [4]).

Disruptions in Notch signaling in a variety of different vertebrate models have been shown to cause alterations in sensory patterning, supporting the lateral inhibitory model in the ear [5]–[9]. Based on studies from Drosophila [10], a model of lateral inhibition in the ear mediated by Notch signaling has emerged in which cells developing as the primary cell type (the hair cell) express a Notch ligand and activate Notch in the surrounding cells, thereby inhibiting them from adopting the same cell fate. These surrounding cells will instead adopt the secondary cell fate, in this case the supporting cell fate [11], [12]. This traditional model of lateral inhibition supports a role for Notch in inhibiting the primary cell fate, but indicates no instructive role in the secondary cell fate. This idea was challenged a number of years ago in the vertebrate central nervous system, in which it was shown that Notch can play an instructive role in the glial cell fate [13]–[16]. For example, expression of an activated form of Notch in the retina leads to an increase in cells expressing Müller glial markers [13]. Similarly in the forebrain Notch promotes the acquisition of a radial glial phenotype [14]; while in the cerebellum, loss of a novel Notch ligand (DNER) or Jagged1 leads to defects in Bergmann glial differentiation [15], [16]. However, whether Notch can play an instructive role in non-glial cell fates, such as the supporting cells of the inner ear, is not known.

Here, to test the role of Notch activation in supporting cell differentiation, we expressed an activated form of the receptor (NICD) in early differentiating hair cells to determine whether Notch signaling can (1) prevent the adoption of the hair cell fate and (2) promote the adoption of the supporting cell fate. Our results show that activation of Notch in differentiating hair cells leads to profound deafness. Histologically, the auditory hair cells shut off a number of different hair cell markers and the inner hair cells lose their characteristic morphology. Specifically, the NICD-expressing inner hair cells gradually adopt a more supporting cell-like morphology and express several supporting cell markers. In addition, our data demonstrates that these effects are not mediated by SOX2, a gene expressed in supporting cells that is upregulated by activating Notch. These results show that Notch actively promotes the supporting cell phenotype in addition to suppressing sensory hair cell marker expression, and that these effects are not dependent upon SOX2 expression.

## Materials and Methods

### Mice

The mouse strains used were as follows: *Gfi1-Cre* [17], *ROSA26-NICD* [18], *ROSA26-LacZ* [19], *ROSA26-SOX2* [20]. The day of birth was considered postnatal day (P)0. This study was carried out in strict accordance with the recommendations in the Guide for the Care and Use of Laboratory Animals of the National Institutes of Health. The animal protocol was approved by the University Committee of Animal Resources (UCAR Protocol No. 101414) at the University of Rochester.

### Histology

#### Paraffin sections

The inner ear was dissected from postnatal mice, fixed in 4% paraformaldehyde overnight and washed in PBS. Ears were then decalcified in 0.2M EDTA (pH 7.3) for 14 days. The tissue was dehydrated through serial EtOH washes from 70% to 100%, cleared in xylene, embedded in paraffin and then sectioned at a thickness of 10 µm.

#### Frozen sections

Heads of P6 mice were bisected, fixed in 4% paraformaldehyde overnight and embedded in Tissue Freezing Medium. Half heads were then cryosectioned at 20 µm.

### Immunohistochemistry

Antigen retrieval was performed on all paraffin sections prior to staining by incubating in 10 mM Sodium Citrate Buffer (pH 6, 0.05% Tween 20) for 20 minutes at 98°C. Sections were incubated in primary antibodies overnight at 4°C, secondary antibodies for 2 hours at room temperature and DAPI nucleic acid stain (1∶24,000) for 8 minutes. The primary antibodies used were as follows: rabbit anti-MYO6 (Proteus Biosciences), rabbit anti-calretinin (Millipore), mouse anti-parvalbumin (Sigma), goat anti-prestin (Santa Cruz), goat anti-SOX2 (Santa Cruz) anti-P27KIP1 (Lab Vision), anti-PROX1 (Chemicon), anti-Na-K-ATPase α1 (Millipore), and anti-β-galactosidase (Abcam).

### Quantification of inner hair cell marker expression

Images of P20 paraffin sections were captured through the mid-modiolar region of the cochlea (ranging between 24 and 36 sections for each mutant or control including roughly equal numbers of basal and apical regions) and relative expression levels of each marker were assessed exclusively in the inner hair cells. Three controls and three mutants were analyzed for each of the hair cell markers (calretinin, parvalbumin and myosinVI). When inner hair cells were brightly and fully stained by the hair cell marker a score of 2 was given (strong), and cells in which hair cell markers were completely negative were assigned a score of 0 (absent). In cases where only a portion of the cell demonstrated signal, or the signal was very low compared to average control expression, these images received a score of 1 (weak).

### Auditory testing

Auditory testing was conducted using a Smart EP Universal Smart Box (Intelligent Hearing Systems). 5 week old mice were anesthetized with an intraperitoneal injection of ketamine (80 mg/kg) in a sterile acepromazine/saline mixture (3 mg/kg). A 10B+ (high frequency transducer/stimulator) probe was placed at the opening to the external auditory meatus. ABR stimuli were 5-ms clicks, or 5-ms tone pips presented at 5 frequencies between 8 and 32 kHz. Stimuli began at 75 dB amplitude and decreased by 5 dB steps to 15–25 dB. 512 sweeps were averaged for each frequency and amplitude. Electrical responses were measured with three subdermal needle electrodes (Grass): one inserted beneath each pinna, and a third, the ground electrode, placed at the vertex. ABR thresholds for a particular frequency were determined by the last visible trace where waves one and two were seen for each stimulus (dB). Wave amplitudes and latencies were determined using IHS software computing capabilities.

To measure DPOAEs, we measured the amplitude of evoked otoacoustic emissions to paired pure tones of frequencies f1 and f2, where f1/f2 = 1.2 and the f1 level was 10 dB above f2. 32 sweeps were made in 5 dB steps starting with f1 at 65 dB and ending at 40 dB. For our equipment, the threshold of DPOAE detection is 3 dB. To calculate the threshold, we used linear regression to fit a line to the points closest to the threshold and calculated the level of f2 necessary for an output of 3 dB.

## Results

### Activation of Notch in early developing hair cells causes deafness

**Figure 1:**
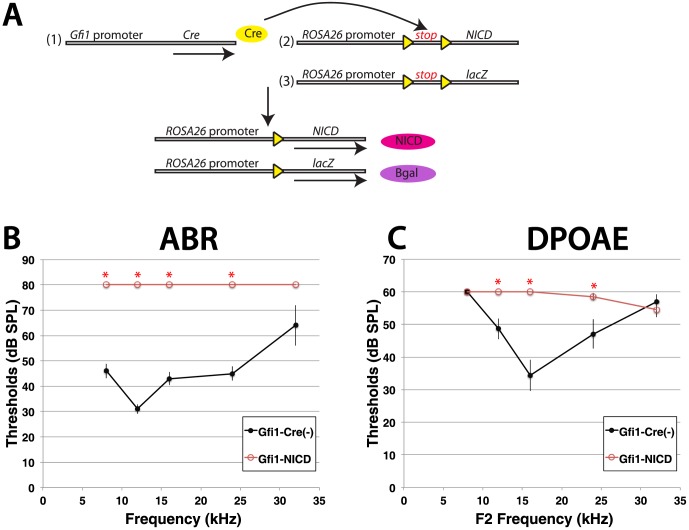
Activation of Notch in differentiating hair cells causes profound deafness. A. Schematic diagram demonstrating constructs for NICD expression in hair cells. (1) Cre is expressed in early developing hair cells under the Gfi1 promoter, causing recombination of a floxed-stop cassette and expression of NICD (2). The cross also contains a ROSA26-lacZ allele, which is similarly recombined upon expression of Cre and marks by lineage the cells expressing NICD (3). B–C. Hearing assessments of Gfi1-NICD mutants and their littermate Cre-negative controls at 5weeks of age. B. Auditory brainstem recordings (ABRs) show that Gfi1-NICD mutants have no responses to the highest sounds levels (80 dB SPL). C. Measurements of distortion product otoacoustic emissions (DPOAEs) also show raised thresholds, but have some responses to sound. To determine significance, a 2-way ANOVA was performed, with pairwise comparisons to determine significance for each frequency, * = p<0.001.

To test the role of Notch during sensory cell differentiation, we generated a mouse model in which activated Notch is expressed in early developing hair cells, allowing us to examine the effects of activated Notch on both hair cell and supporting cell differentiation. To generate this model, we crossed mice expressing Cre under the Gfi1 promoter [17] to mice expressing an activated form of the Notch receptor, NICD (N_otch I_ntrac_ellular D_omain) under the ubiquitous ROSA26 promoter [18] (Fig. 1A). Gfi1 is a transcription factor expressed soon after hair cells begin differentiating in the ear, approximately embryonic (E)13–14 in the vestibular system and E15–16 in the cochlea [17], [21], [22]. The ROSA26-NICD allele contains an intervening floxed-stop cassette, preventing NICD expression unless Cre is present. Offspring containing both a Cre allele and an NICD allele (hereafter referred to as Gfi1-NICD mutants) were born and survived until adulthood. In addition, a ROSA26-lacZ allele [19] was also bred into the cross, so that hair cells expressing NICD would be marked by ß-galactosidase. Upon weaning it was apparent that some of the Gfi1-NICD offspring lacked a Preyer reflex, the ear flick response to sound, indicating a hearing impairment. To investigate this further, we measured auditory brainstem responses (ABRs) and distortion product otoacoustic emissions (DPOAEs) in Gfi1-NICD mutants (n = 8) and their littermate controls (n = 8). Results of the ABRs showed that the Gfi1-NICD mutants showed no responses to even the highest levels of sound (80 dB SPL), indicating profound deafness (Fig. 1B). In addition, measurements of outer hair cell function, DPOAEs, also demonstrated raised thresholds when compared to controls at most frequencies, indicating that outer hair cell function is compromised (Fig. 1C). These results show that expression of activated Notch in early differentiating hair cells affects the function of the organ of Corti.

### Hair cell markers are downregulated in NICD-expressing hair cells and SOX2 is upregulated

**Figure 2:**
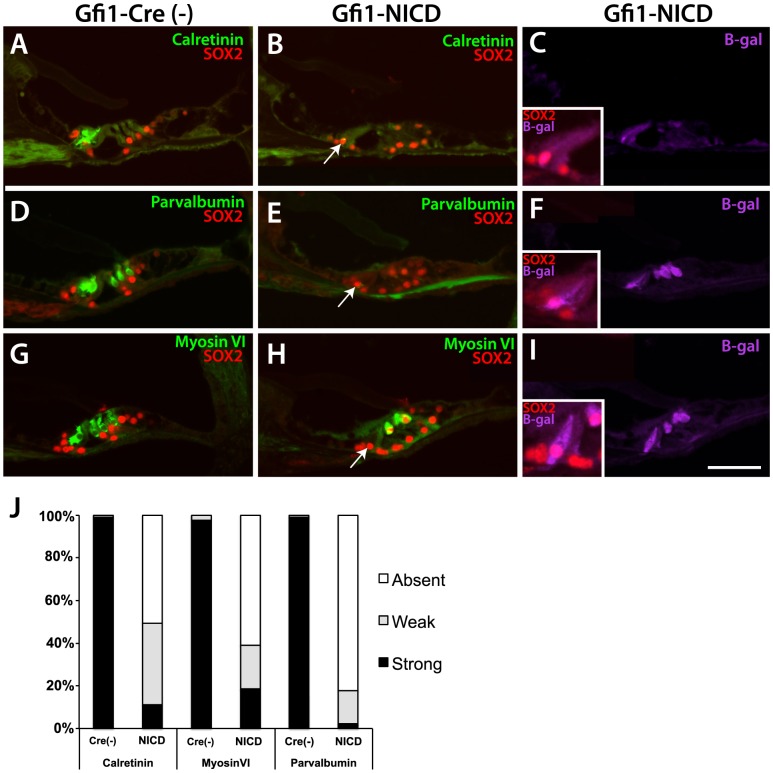
At P20, NICD-expressing hair cells in the cochlea have downregulated hair cell markers. A–I Paraffin sections through the P20 cochlea stained for hair cell markers, SOX2, and ß-galactosidase. A, D, G. Calretinin, parvalbumin, and myosin VI all show expression in the inner and outer hair cells at P20 (although calretinin expression is weak in outer hair cells at P20). B, E, H. Both calretinin and parvalbumin are shut off in the Gfi1-NICD inner and outer hair cells, whereas myosin VI is generally still expressed in the outer hair cells in the mutant (H). Gfi1-NICD inner and outer hair cells express SOX2 and the inner hair cells have a more basally-located nucleus than in controls (B,E,H, arrows and insets in C,F,I showing higher power images of inner hair cells expressing ß-galactosidase and SOX2). J. Quantification of relative expression levels of indicated markers in the Gfi1-NICD (NICD) inner hair cells and littermate controls [Cre(-)]. Scale bar in I = 100 microns ( = 50 microns for inset panels).

To examine the effects of activated Notch on the molecular and histological differentiation of cochlear hair cells, we sectioned postnatal day (P)20 Gfi1-NICD cochleae and littermate controls and examined the expression of several hair cell markers by immunohistochemistry. Results of these studies showed that a number of established hair cell markers, including calretinin, parvalbumin, and myosin VI, were either not expressed in the hair cells or were severely downregulated when compared to controls (Fig. 2). In the case of calretinin and parvalbumin, the majority of both inner and outer hair cells did not express these markers (Fig. 2A–F). In the case of myosin VI, while the majority of inner hair cells downregulated this marker, the outer hair cells largely maintained expression of myosin VI (Fig. 2G–I). The absence of hair cell markers was not due to cell loss, as the cells still expressed ß-galactosidase (Fig. 2C,F,I). Inner hair cell marker expression was quantified for each marker (myosin VI, calretinin, and parvalbumin) in three Gfi1-NICD mutant cochleae and controls (Fig. 2J). Sections through the mid-modiolar regions of the cochlea were scored for presence (black regions of bars) or absence of the marker signal (white). If the marker was only partially expressed within the cell, or was very weak, those inner hair cells were scored as weak (gray area of bars). As expected, the control sections showed greater than 95% strong expression, with only a few inner hair cells exhibiting weak expression, and no control sections showing absent expression. In contrast, at least 50% of the mutant sections showed no expression of the marker in the inner hair cells (for parvalbumin it was greater than 80%). Even when the marker wasn't completely absent, weak expression was observed in 15–40% of the inner hair cells in the mutant cochlea, whereas weak expression constituted only 3–4% of the control inner hair cells. These results show that greater than 80% of the hair cells expressing NICD have either downregulated or completely shut off hair cell-specific markers by P20.

In addition to the downregulation of the hair cell markers, the NICD-expressing hair cells upregulated the HMG transcription factor SOX2 (Fig. 2), a progenitor and supporting cell marker that is normally downregulated in auditory hair cells early postnatally. As expected SOX2 was not detectable in control hair cells at P20 (Fig. 2A,D,G), but was apparent in the Gfi1-NICD inner and outer hair cells (Fig. 2B,E,H). Interestingly, as SOX2 is a transcription factor localized to the nucleus, it was apparent that the nuclei of the NICD-expressing inner hair cells were more basally positioned than in control inner hair cells (Fig. 2B,E,H, arrows; insets in C,F, and I). This nuclear position is consistent with the position of the nucleus in supporting cells rather than hair cells, where it is more apically situated.

**Figure 3:**
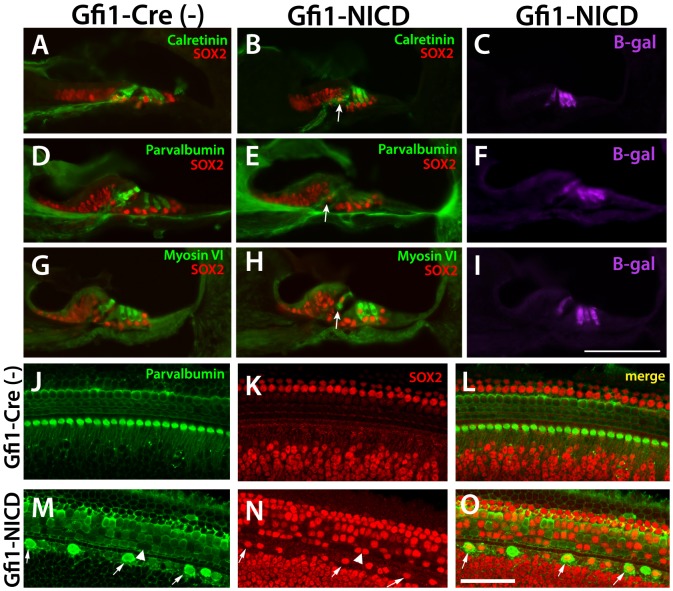
At P6, Some hair cell markers are downregulated in the inner hair cells. A–I. Frozen sections through the P6 cochlea stained for hair cell markers, SOX2, and ß-galactosidase. A, D, G. Calretinin, parvalbumin, and myosin VI all show expression in the inner and outer hair cells at P6 (although parvalbumin expression is weaker in outer hair cells at P6). B, E, H. Both calretinin and parvalbumin are downregulated in the Gfi-NICD inner hair cells, although outer hair cell expression is largely maintained. Interestingly, myosin VI (G–I) is generally indistinguishable from the controls at this time point. Scale bar in I = 100microns. J–O. Wholemount cochlea stained for hair cell (parvalbumin) and supporting cell (SOX2) markers. Many of the inner hair cells have shut off parvalbumin and upregulated SOX2 by this time point (M–O, arrows). A few outer hair cells have downregulated parvalbumin (M–O, arrowhead), but most have upregulated SOX2.

To determine how early these markers were shut off, we examined Gfi1-NICD cochleae and littermate controls at P6. Results of these experiments showed that many inner hair cells did not express either calretinin or parvalbumin, similar to results at P20, whereas the outer hair cells largely still expressed these markers (Fig. 3A–F). Interestingly, the majority of both inner and outer hair cells continued to express myosin VI (Fig. 3G–I), indicating that not all hair cell markers were downregulated at the same time. As at P20, the hair cells also expressed SOX2, although unlike P20, the NICD-expressing inner hair cell nuclei were normally positioned at this time. To get a better idea of how many hair cells had begun downregulating hair cell markers and had upregulated SOX2, we examined Gfi1-NICD cochleae and their littermate controls in wholemount (Fig. 3J–O). These results showed that a majority of the Gfi1-NICD inner hair cells had downregulated parvalbumin and upregulated SOX2, whereas the majority of the outer hair cells still expressed parvalbumin, although there were variations in the levels of expression (Fig. 3M).

### NICD-expressing hair cells demonstrate an abnormal morphology resembling supporting cells, and upregulate supporting cell markers

**Figure 4:**
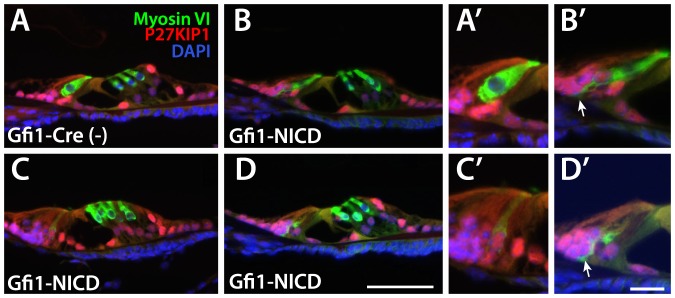
NICD-expressing inner hair cells show morphology changes and abnormal P27KIP1 expression at P11. A–D. Paraffin sections from Gfi1-NICD cochlea and littermate controls at P11. A′-D′ show higher power views of the inner hair cells, which demonstrate morphological and molecular changes due to Notch activation. The changes in the inner hair cells in the Gfi1-NICD mutants (B,C,D and B′C′,D,) include weaker MYO6 expression, lack of the normal flask-like shape, more basally positioned nulcei, and nuclear P27KIP1 expression normally only observed in supporting cells at this time. Scale bar in D = 100 microns for A–D, and in D′ = 25 microns for A′–D′.

To further investigate the morphological and molecular changes taking place in the cochlear hair cells, we examined Gfi1-NICD cochlea and controls at P11, using both myosin VI and P27KIP1, a nuclear cell cycle inhibitor expressed in postnatal supporting cells. Results of these studies showed that although most of the inner hair cells appeared to have a normal morphology and maintained expression of myosin VI, some of the inner hair cells had begun downregulating myosin VI and displayed an abnormal hair cell morphology (Fig. 4). Specifically, some of the NICD-expressing inner hair cells had lost their flask-like shape, displayed a more basally-positioned nucleus, and demonstrated processes that contacted the basement membrane (Fig. 4A′–D′). These features are consistent with a supporting cell morphology.

**Figure 5:**
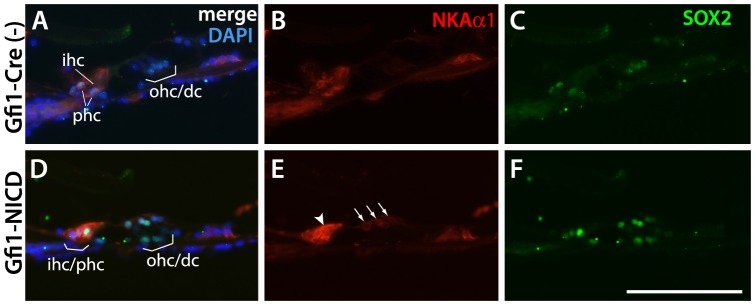
The mature supporting cell marker NKAα1 is upregulated in NICD-expressing inner and outer hair cells. A–F. Paraffin sections from Gfi1-NICD and Cre-negative control cochleae at P20. A–C. Control sections showing the normal expression of NKAα1 in the phalangeal cells (phc) surrounding the inner hair cells (ihc), which are also coexpressed with SOX2. Deiter's cells also show expression of SOX2, although not NKAα1. D–F. Gfi1-NICD expressing inner hair cell has upregulated NKAα1 (arrowhead). Surprisingly, the NICD-expressing outer hair cells (ohc) have also upregulated NKAα1, although much more weakly than in the inner hair cells. Both inner and outer hair cells also show upregulation of SOX2. Scale bar = 100 microns.

As many of the supporting cell markers examined were also progenitor cell markers that eventually were downregulated in the supporting cells, we were interested in whether the NICD-expressing hair cells expressed any mature supporting cell markers. Since the inner hair cells showed the most dramatic cell morphological and molecular changes in response to NICD expression, we reasoned that these cells may be converting to inner phalangeal cells, the supporting cells that surround the inner hair cells. We therefore examined expression of the Na-K-ATPase α1 subunit (NKAα1) [23], [24], an inner phalangeal cell marker, in P20 cochlea from Gfi1-NICD mutants and their littermate controls (Fig. 5). Results showed that NKAα1 was upregulated dramatically not only in the NICD-expressing inner hair cells (Fig. 5D–F), but also more mildly in the outer hair cells (Fig. 5E; arrowhead and arrows). These results demonstrate that the NICD-expressing hair cells express mature supporting cell markers in the adult cochlea, indicating that Notch promotes a supporting cell phenotype, and not simply a progenitor cell phenotype.

**Figure 6:**
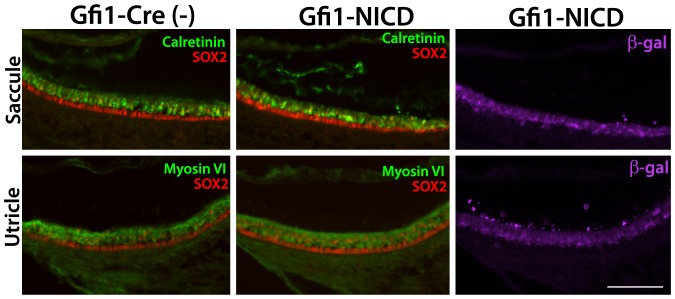
NICD-expressing hair cells exhibit expected markers in the vestibular regions. A–F . Frozen sections through the vestibular regions of P6 inner ears. Hair cell markers (calretinin and Myosin VI; B and E) are expressed similarly to controls (A and D) in the indicated regions of the vestibular system. Sections were co-labeled with ß-galactosidase expression, which is shown in a different panel (C, F) to better show the expression. Scale bar in F = 100 microns.

Interestingly, although the Gfi1-NICD mutants were profoundly deaf, the mice did not demonstrate any classic behaviors indicative of a vestibular defect, including circling or head-shaking behavior. To investigate this further, we examined the utricular and saccular maculae in Gfi1-NICD mutants to determine whether there were any histological or molecular changes taking place. These studies showed no downregulation of hair cell markers, or changes in cell morphology (Fig. 6), indicating that surprisingly, Notch does not have the same effects on vestibular hair cells that it has on the auditory hair cells.

### Activation of SOX2 in developing hair cells does not recapitulate the effects of NICD overexpression

**Figure 7:**
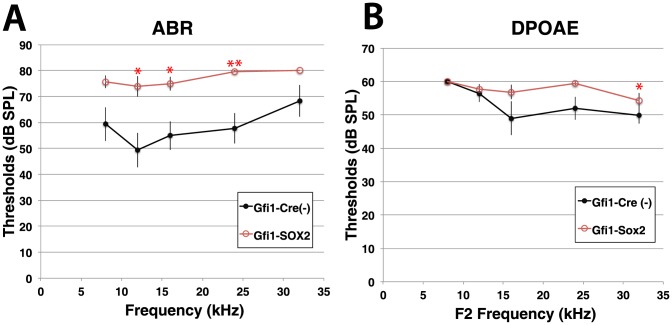
Auditory testing of Gfi1-SOX2 mutants demonstrates that SOX2 expression causes hearing impairment. A. Auditory brainstem recordings (ABRs, left) of Gfi1-SOX2 mutants and littermate controls at 5 weeks of age show that SOX2-expressing mice have significantly raised thresholds compared to controls. B. Measurements of distortion product otoacoustic emissions (DPOAEs) of the same mice tested for ABR, also show raised thresholds at 5 weeks. To determine significance, a 2-way ANOVA was performed, with pairwise comparisons to determine significance for each frequency: ** = p<0.001, * = p<0.05.

Given that activation of Notch led to an upregulation of SOX2, and SOX2 is expressed in supporting cell of the inner ear, we were interested in determining whether SOX2 was mediating any of the changes observed in the NICD-expressing hair cells. To accomplish this, we used an experimental paradigm similar to the Notch activation and crossed Gfi1-Cre to mice expressing SOX2 under the ubiquitous ROSA26 promoter [20]. We tested whether expression of SOX2 has any effects on hearing using ABR and DPOAE analysis. Results of these tests showed that Gfi1-SOX2 mutants (n = 14) were hearing impaired when compared to littermate controls (n = 9), although they were not as profoundly deaf as the Gfi1-NICD mutants (Fig. 7). Specifically, whereas Gfi1-NICD mutants showed no responses at even the highest levels of sound tested, Gfi1-SOX2 mutants demonstrated some responses, although at most frequencies their thresholds were significantly raised (Fig. 7A). Similarly DPOAE responses were also detected in Gfi1-SOX2 animals, although again these responses showed increased thresholds when compared to controls (Fig. 7B), although at most frequencies these increases were not significantly different. These data indicate that both inner and outer hair cells are functionally compromised by SOX2 overexpression, but the effects are not as severe as those produced by NICD overexpression.

**Figure 8:**
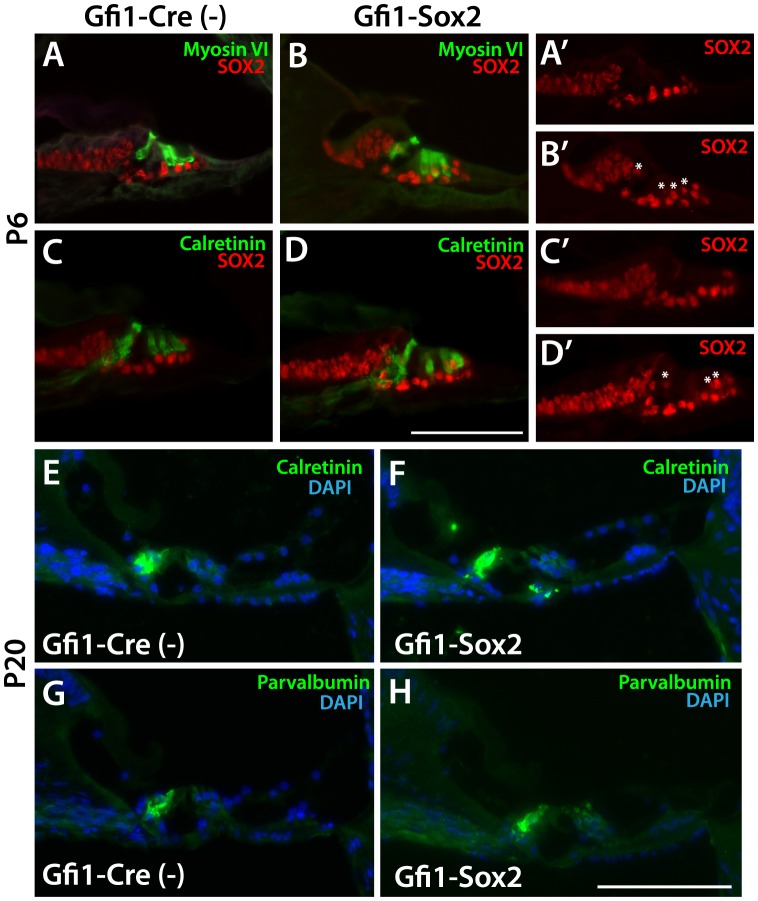
Histological assessment of SOX2-expressing hair cells shows no morphological changes or downregulation of hair cell markers over time. A–D. Both calretinin and myosin VI are expressed normally in Gfi1-SOX2 mutants (B,D) at P6 when compared to controls (A,C). A′–D′. Expression of SOX2 alone (shown also in A–D) demonstrates the upregulation of SOX2 in the inner and outer hair cells in Gfi1-SOX2 mutants (asterisks indicate hair cell nuclei expressing SOX2 in B′–D′). Scale bar in D = 100 microns for A–D. E–H. Expression of indicated hair cells markers in Gfi1-SOX2 mutants (F,H) is maintained normally at P20 when compared to controls (E,G). Scale bar in H = 100 microns for E–H.

To determine the effects of ectopic SOX2 expression on the molecular and histological aspects of sensory cell differentiation, we examined hair cell and supporting cell markers by immunohistochemistry. Surprisingly, these results showed that hair cell markers were maintained normally in SOX2-expressing cells (Fig. 8). No differences were detected in expression of myosin VI or calretinin at P6 (Fig. 8 A–D). Even at P20, no downregulation of either calretinin or parvalbumin was observed in the inner hair cells (Fig. 8E–H), although in most cases neither marker was detectable at this time in Gfi1-NICD expressing hair cells (Fig. 2). Moreover, no differences were detected in the morphology of the SOX2-expressing hair cells. Specifically, we did not observe any hair cells in contact with the basement membrane, and the hair cell nuclei retained their normal apical position. These data show that while SOX2 expression may have some effects on the function of the hair cell, SOX2 expression does not interfere with hair cell gene expression or morphology, indicating that SOX2 does not mediate the effects of Notch in repressing the hair cell phenotype while promoting a supporting cell phenotype.

## Discussion

Our results indicate that Notch plays an important role in suppressing hair cell gene expression in differentiating supporting cells. In addition, we have shown that Notch actively promotes the expression of a number of supporting cell markers. This change in gene expression is accompanied by changes in cell morphology, particularly in the inner hair cells, in which cells lose their characteristic flask-like shape, show contact with the basement membrane, and have a more basally-positioned nucleus, features consistent with a supporting cell fate. This instructive role in the supporting cell differentiation is similar to the role that Notch plays in the CNS in promoting the glial cell fate [13]–[16]. Not surprisingly, this switch in gene expression leads to severe consequences for hair cell function, as the Gfi1-NICD mutants were profoundly deaf. We also examined whether these effects were mediated by SOX2, a likely downstream target of Notch signaling [25]–[28] expressed in developing and mature supporting cells [29], [30]. Interestingly, although SOX2 expression interferes with hair cell function to some degree, we did not observe the same molecular and cell morphology changes in the Gfi1-SOX2 mutants that were seen in the NICD-expressing mutants.

The protracted and gradual nature of the expression and morphology changes in the cochlear hair cells after NICD expression was unexpected. Although we began to express NICD during early hair cell differentiation (E15–E16.5 in the cochlea [17]), widespread changes in gene expression and morphology were not observed until P20, although some downregulation of hair cell markers could be observed at P6. A possible reason for this slow change in phenotype is that although NICD may inhibit expression of hair cell markers immediately through suppression of transcription, ultimately loss of the hair cell markers may depend on the half-life of the protein. Indeed, this may explain why some markers are downregulated more quickly than others (Fig. 2J). Myosin VI, for example, seemed particularly resistant to downregulation compared to calretinin or parvalbumin, as expression was frequently observed at P6 while the other two markers were largely absent (Fig. 3). It also appeared that in some cases, downregulation of hair cell-specific marker expression was required before the cells could express supporting cell markers, or show changes in morphology. For example at P11, upregulation of P27KIP1 and contact with the basement membrane was only observed in a subset of inner hair cells that had significantly downregulated myosin VI (Fig. 4). Thus, suppression of hair cell genes may be a required step for the proper expression of supporting cell genes.

Another surprising result was the differential effects of Notch activation depending on the type of hair cell. For example, while we saw dramatic changes in gene expression, morphology and function in inner hair cells and milder changes in outer hair cells, there were no apparent effects on the vestibular hair cells. The reasons for this are currently unclear, although it suggests that Notch or its targets are regulated differentially depending on the type of hair cell. In this context, it is interesting to note that some adult vestibular hair cells (likely type II) continue to express SOX2, which is exclusively restricted to supporting cells in the cochlea [30], [31]. However, control of SOX2 function may be regulated by aspects other than transcriptional control, including potentially other SoxB members such as SOX21. It is interesting to note that SOX21 is also differentially expressed between the cochlear and vestibular regions in the chicken, and overexpression of SOX21 results in a bias towards the hair cell fate in vestibular regions but not in the cochlea (basilar papilla) [32]. It is also possible that the timing of Gfi1-Cre expression is slightly different in different types of hair cells, and this may account for the differential effects. Our results indicate that activated Notch and SOX2 must be shut off in the hair cells of the cochlea for normal hair cell function, but similar downregulation may not be required for vestibular hair cells.

Our results are in contrast to those of Liu et al., [33], who used the *Atoh1^CreER^* to express NICD in developing hair cells, and found no changes in hair cell gene expression, hair cell function or morphology, despite upregulation of SOX2. Although these two Cre alleles have slightly different times of induction, with *Atoh1^CreER^* being slightly earlier than the Gfi1-Cre used in our study [22], we consider it more likely that these differing outcomes are due to the different endpoints examined in each study. For example Lui et al., [33] looked at changes in hair cell gene expression at E19, whereas our study looked at later postnatal ages. Indeed, as discussed above, we demonstrated that there are only mild gene expression changes beginning around P6, which do not become pronounced until P20, along with morphological changes. In addition to the earlier induction, Lui et al., [33] also induced Cre at later time periods, including P0 and P1, to look at the effects of later NICD re-activation in developing and adult hair cells. Similar to their results at the earlier time points, they observed no changes in hair cell gene expression, morphology, or function (as assessed by FM 4–64FX dye) even at adult stages. However, it appeared that very little ectopic SOX2 expression was detected in the inner hair cells, indicating that significant NICD activation was not achieved in this cell type, since in our experience SOX2 expression is an excellent readout for NICD activation [25], [34]. Thus, since the major effects that we observed were in the inner hair cells, it is possible that they saw few effects on the cochlea due to the low recombination rates of the NICD allele in the inner hair cell population. However, it is also possible that NICD overexpression at P0–P1 is too late to induce cell fate changes in the hair cells [33]. Therefore, it remains an open question as to whether Notch can induce supporting cell-like changes in postnatal and adult inner hair cells.

Our results show that Notch activation leads to suppression of hair cell markers and an upregulation of supporting cell markers in cells that have begun differentiating as hair cells. These results indicate that Notch not only prevents the hair cell fate, but can promote a supporting cell fate, similar to its role in promoting certain glial cell phenotypes in the central nervous system [13]–[16]. It is interesting to note that the glial cell types in which Notch has been shown to play an instructive role (including radial glia and particular radial glial subtypes including Bergman and Müller glia) can also act as progenitors [35], [36]. It will be interesting to determine if there are further similarities between supporting cells and radial glia subtypes. Our data also shows that, in contrast to a previous study [33], auditory hair cells are not irreversibly specified at embryonic time points, and have the capability of acquiring aspects of the supporting cell phenotype. Our results have important implications for those interested in regenerating sensory region cell types in the inner ear. Specifically, our data indicates that activation of Notch is an important step in the specification of supporting cells, whereas suppression of Notch activation appears to be a required step in the differentiation of auditory hair cells.
